# Enteroendocrine cell regulation of the gut-brain axis

**DOI:** 10.3389/fnins.2023.1272955

**Published:** 2023-11-07

**Authors:** Joshua R. Barton, Annie K. Londregan, Tyler D. Alexander, Ariana A. Entezari, Manuel Covarrubias, Scott A. Waldman

**Affiliations:** ^1^Department of Pharmacology, Physiology, and Cancer Biology, Thomas Jefferson University, Philadelphia, PA, United States; ^2^Department of Neurosciences, Thomas Jefferson University, Philadelphia, PA, United States; ^3^Sidney Kimmel Cancer Center, Thomas Jefferson University, Philadelphia, PA, United States

**Keywords:** gut-brain axis, enteroendocrine cells, neuropod cells, irritable bowel syndrome (IBS), semaglutide, GLP-1 analogues, linaclotide, GUCY2C

## Abstract

Enteroendocrine cells (EECs) are an essential interface between the gut and brain that communicate signals about nutrients, pain, and even information from our microbiome. EECs are hormone-producing cells expressed throughout the gastrointestinal epithelium and have been leveraged by pharmaceuticals like semaglutide (Ozempic, Wegovy), terzepatide (Mounjaro), and retatrutide (Phase 2) for diabetes and weight control, and linaclotide (Linzess) to treat irritable bowel syndrome (IBS) and visceral pain. This review focuses on role of intestinal EECs to communicate signals from the gut lumen to the brain. Canonically, EECs communicate information about the intestinal environment through a variety of hormones, dividing EECs into separate classes based on the hormone each cell type secretes. Recent studies have revealed more diverse hormone profiles and communication modalities for EECs including direct synaptic communication with peripheral neurons. EECs known as neuropod cells rapidly relay signals from gut to brain via a direct communication with vagal and primary sensory neurons. Further, this review discusses the complex information processing machinery within EECs, including receptors that transduce intraluminal signals and the ion channel complement that govern initiation and propagation of these signals. Deeper understanding of EEC physiology is necessary to safely treat devastating and pervasive conditions like irritable bowel syndrome and obesity.

## 1. Introduction

Our viscera are in constant conversation with our peripheral and central nervous system, a continuous two-way surveillance termed “interoception” (Maniscalco and Rinaman, 2018). This perception of our internal functions, while mostly unconscious, manifests itself in our behavior: influencing mood, activity level, and motivation (Büttiker et al., 2021). The concept of interoception has been discussed academically for over a century, notably championed by physiologist Carl Lange and psychologist William James, who wrote in *Principles of Psychology* (James, 1918):

“If our hypothesis is true, it makes us realize more deeply than ever how much our mental life is knit up with our corporeal frame, in the strictest sense of the term. Rapture, love, ambition, indignation, and pride, considered as feelings, are fruits of the same soil with the grossest bodily sensations of pleasure and pain.”

Less esoterically, interoception serves to maintain homeostasis: the function and faculties of tissues are reported with afferent signals, which instruct compensatory responses through efferent signals from the central nervous system (Chen et al., 2021). With respect to the gastrointestinal (GI) tract, this cross-talk takes place along the gut-brain axis, and comprises signals sent directly through neurons as well as indirectly through endocrine hormones (Clemmensen et al., 2017; Gershon and Margolis, 2021). Classically, the gut-brain axis encodes the nutritive information of luminal contents as well as motility and distention (Forum, 2015; Enck et al., 2017). Recent studies have demonstrated the far broader influence of the gut on the brain and vice versa regulating microglial proliferation, T cell trafficking and inflammation, insulin resistance, amyloid deposition, and synapse formation (Heijtz et al., 2011; Erny et al., 2015; Kasubuchi et al., 2015; Shreiner et al., 2015; Benakis et al., 2016; Perry et al., 2016; Wang et al., 2017). Special attention has been given to the influence of the microbiota on the gut-brain axis, in large part due to the advent of tools to study the diversity of commensal organisms within the intestine (Kennedy et al., 2018). The interdependence of the brain on signals from the gut is further underscored by studies in germ free mice, which have abnormal brain structure and function correlated with the absence of a postnatal microbiota (Jena et al., 2020).

## 2. Enteroendocrine cells: gut interoceptors

The surface area of our digestive tract measures, on average, 32 m^2^, comprising the largest interface our bodies have with the “outside” world (Helander and Fändriks, 2014). The small intestine alone comprises 30 m^2^ of surface area, expanded by finger-like projections (villi), most of which are comprised of enterocytes: cells designed to absorb, secrete, and provide a barrier between luminal contents and the rest of the body (Elmentaite et al., 2021). This barrier function requires that intestinal cells are continually sloughed off into the lumen and replacement cells are constantly produced, resulting in complete turnover of intestinal villi every 2-5 days (Creamer et al., 1961). All cells in the intestinal epithelium arise from intestinal crypts, which harbor stem cells characterized by the expression of Lgr5 (Sato et al., 2009). The crypt-villus structure of the intestine maximizes surface area, while maintaining a protected stem cell niche, and is conserved across all mammals except for the platypus, who forgoes villi for large surface folds of pseudostratified epithelium (Krause, 1975). At least seven different lineages of intestinal cells differentiate from the stem cell niche: enterocytes (absorption and barrier function), goblet cells (mucus-producing), Paneth cells (secrete antimicrobial peptides and growth factors to the crypt), microfold cells (maintain and communicate with lymphoid follicles), cup cells (unknown function), tuft cells (antigen presentation), and EECs (hormone release and gut-brain communication) (Gerbe et al., 2012; Gerbe and Jay, 2016).

Of the specialized intestinal epithelial cells, EECs are those responsible for communicating signals from gut to brain and other organs. The hierarchy of differentiation factors necessary for an intestinal stem cell to become an EEC are well established. Three transcription factors primarily drive a stem cell to an EEC fate: Math1 (Atoh1) first drives the cell toward a secretory lineage (as opposed to an absorptive lineage), followed by Neurogenin3 expression leading the cells to become mature, hormone-producing epithelial cells (Yang et al., 2001; Jenny et al., 2002). Finally, Neurogenin3 induces NeuroD1 expression, which drive cells to produce secretin and function as endocrine cells (Mutoh et al., 1997). EECs are canonically classified by their hormone expression, which they release basally into vasculature in the lamina propria (Beumer et al., 2020). As EEC hormones were discovered in the blood and then the intestine, they were named by a now inscrutable letter code. Enterochromaffin (EC) cells, which we now know to produce serotonin (5-HT), were discovered and named by their reactivity with chrome salts (the chromaffin reaction) (Heidenhain, 1870; Vialli and Erspamer, 1933). Later, additional types of EECs were classified by the size of vesicles stored within them: Small (S cells, secretin), Intermediate (I cells, Cholecystokinin, CCK), Large (L cells, Glucagon-Like Peptide 1, GLP1 and Peptide YY, PYY), and Large-like (K cells, Glucose-dependent insulinotropic polypeptide, GIP) (Fothergill and Furness, 2018). Two more groups of EECs were defined by their morphological similarity to pancreatic cells, and named A cells (ghrelin, GHRL) and D cells (Somatostatin, Sst) (Bordi et al., 2000). Still another naming convention classified more EECs by their immunoreactivity to hormone antibodies, M cells (Motilin), N cells (Neurotensin, Nts), S cells (Secretin, Sct), and X cells (*Also Ghrelin*, meaning that Ghrelin characterizes both A and X cells) (Rehfeld, 2012). Unsurprisingly, this nomenclature has resulted in ample confusion and inconsistency in the EEC field, as well as incorrectly ascribing the concept of “one cell-one hormone” to EECs (Helander and Fändriks, 2012). Through elegant studies in mouse and organoid models and high-throughput sequencing, we now know that EECs contain multiple hormones and are directed spatially and temporally to express different hormone suites by extracellular signals (Habib et al., 2012; Beumer et al., 2018, 2020). Using novel mouse models which express fluorescent proteins with well-defined half-lives in EECs, studies have revealed the precise transcriptional processes that transform a developing epithelial cell into a mature EEC (Gehart et al., 2019). Together, these studies have revealed the gene expression profiles defining subtypes of EECs, which provide a far more accurate picture of what defines different EEC lineages. Highlighting the “endocrine” nature of EECs, all EEC hormones signal from gut to brain, and stimulate receptors located in circumventricular brain nuclei (not blocked by the blood brain barrier) (Table 1). For example, to signal emesis after ingestion of a potentially harmful substance, 5-HT is released from ECs into the bloodstream, traveling to the area postrema of the nucleus tractus solitarius to induce vomiting (Hagbom et al., 2011).

**Table 1:** Enteroendocrine nomenclature.

Cell name	Hormone	Hormone receptor	Receptor expression by circumventricular brain region
D cell	Somatostatin (SST)	SSTR1, SSTR2	Hypothalamus (Breder et al., 1992)
EC (enterochromaffin) cell	Serotonin (5-HT)	5-HT_1-7_	Hypothalamus (Burnet et al., 1995), area postrema (Glaum et al., 1992)
I cell	Cholecystokinin (CCK)	CCK1 (Alimentary Tract), CCK2 (Brain)	Hypothalamus and area postrema (Moran et al., 1986)
K cell	Glucose-dependent Insulino-Peptide (GIP)	GIPR	Hypothalamus (ARC) (Adriaenssens et al., 2019)
L cell	Glucagon-Like Peptide 1 (GLP1)	GLP-1R	Hypothalamus, NTS, area postrema (Sisley et al., 2014)
	Peptide YY (PYY)	Y1R, Y2R (most selective), Y5R	Hypothalamus (Batterham and Bloom, 2003), area postrema (Deng et al., 2001)
M cell	Motilin	MLN-R	Hypothalamus (Depoortere et al., 1997)
N cell	Neurotensin (NTS)	NTSR1 (neurons), NTSR2 (glia), NTSR3 (intracellular)	Hypothalamus (Tanaka et al., 1990)
S cell	Secretin (SCT)	SCT-R	Hypothalamus (Siu et al., 2006)
X and A cell	Ghrelin (GHRL)	GHRL-R	Hypothalamus, NTS, and area postrema (Zigman et al., 2006)

### 2.1. Neuropod cells

Originally, EECs were thought to communicate with the brain solely through hormonal means (Ganong, 2000). Some signals, however, have been shown to travel more quickly than endocrine signals could be sent from gut to brain. One study demonstrated that satiety-associated (AgRP+) neurons in the arcuate nucleus are activated less than 30 seconds after nutrient infusion (Su et al., 2017). Another study showed that, while satiety hormones CCK and PYY induce acute reaction of AgRP+ neurons after infusion, these effects take on the order of minutes, not seconds, to induce neuronal changes (Beutler et al., 2017).

Mounting evidence reveals that EECs also communicate shorter distances through paracrine signaling with peripheral nerves, especially the vagus nerve (Schwartz and Moran, 1994; Dockray, 2013). Terminal fibers of the vagus nerve can be found throughout the GI tract and even extend to the apical tips of villi (Powley et al., 2011). These fibers express receptors for multiple EEC hormones, and can serve as peripheral receivers of EEC signals (Egerod et al., 2018). The hormone CCK has been particularly well-studied in this context, an interaction which is primarily a paracrine signal of CCK from EECs to CCK1 Receptors (CCK1R) on vagal afferents (Whited et al., 2006; Raybould, 2007). The interaction of CCK with vagal afferents induces satiety (synergistically with leptin), decreases gastric motility, and inhibits hepatic glucose production via a gut-brain-liver neuronal axis (Peters et al., 2006; Whited et al., 2006; Cheung et al., 2009). Elegant pharmacologic studies infusing Protein Kinase A (PKA) inhibitors into the duodenum revealed that CCK1R activity in vagal afferents is PKA dependent, and that CCK’s action on glucose production requires PKA activity in these neurons (Rasmussen et al., 2012). Conversely, ghrelin exerts the opposite effect on the gut-brain-liver axis, decreasing PKA activity in vagal afferents and increasing hepatic glucose production (Lin et al., 2019). The effects of PYY and GLP-1 on food intake have also been shown to require an intact vagus nerve (Abbott et al., 2005). Though the site of action of GLP-1 agonists is an area of ongoing study (see section 6.1), knockdown and knockout studies of GLP-1 Receptors (GLP-1R) reveal that the glucose-controlling effects of GLP-1 are reliant on GLP-1R expression in peripheral neurons (Varin et al., 2019; Brierley and de Lartigue, 2022). Further, truncally vagotomized humans did not respond to GLP-1R agonists with decreased food intake, gastric emptying, glucagon secretion, or increased insulin secretion (Plamboeck et al., 2013). Thus in humans and in animal models, EECs exhibit profound effects on vagal signaling through paracrine hormone signaling.

Some studies imply that rapid signaling, on the order of seconds, from gut to brain is caused by direct synaptic connection from EECs to vagal neurons, signaling not by CCK or PYY, but through glutamate (Kaelberer et al., 2018). These specialized EECs, called neuropod cells, were first identified in PYY-GFP mice as exhibiting long basal processes that extended into the lamina propria (Bohórquez et al., 2011). Electron microscopy of these basal pseudopods revealed that these axon-like processes were “escorted” by enteric glia, and that the processes could be enhanced in organoid culture by neuronal and glial growth factors (Bohórquez et al., 2014). Rabies tracing from CCK+ and PYY+ EECs revealed that these pseudopods formed direct synaptic connections with peripheral neurons, and that these connections could be recapitulated in vitro (Bohórquez et al., 2015). Further, it has now been shown that these neuropod cells can be targeted by optogenetic means, and encode not only satiety signals, but taste preference between sugar and artificial sweeteners (Buchanan et al., 2022). Neuropod cells communicate efferent and afferent signals, express presynaptic and postsynaptic proteins, and communicate with afferent neurons through 5-HT and efferent neurons through norepinephrine (Bellono et al., 2017). These studies have revealed a unique and direct gut-brain connection that can communicate nutritive and noxious signals in real time directly to peripheral neurons. Thus, neuropod cells are not defined by a specific hormone (e.g., CCK, serotonin), but by their formation of basal pseudopods that communicate synaptically with peripheral neurons. In that context, while a portion of CCK-, PYY-, and 5-HT-positive EECs have neuropod cell properties, neuropod cells are a separate subtype of EEC without a singular, definitive protein marker. Though exciting, the field of “neuropod cell” biology is relatively new and based on a comparatively limited scope of publications. Future studies elucidating the role of the neuropod in its interaction with axons and glia are necessary to contextualize the importance of synaptic-vs-paracrine signaling from gut to brain.

### 2.2. Synaptic signaling by neuropod cells

As neuroendocrine cells, neuropod cells are filled with neuropeptides. Neuropeptides are “small proteinaceous substances produced and released by neurons through the regulated secretory route and acting on neural substrates” (Russo, 2017), a definition which separates neuropeptides from peptide hormones on the basis of being released by neurons. However, neuropod cells straddle the line between neuron and endocrine cell, thus peptide hormones found within neuropod cells are more properly defined by whether the secreted peptide is communicating through endocrine or synaptic means (Kaelberer et al., 2020). The hyperexcitability of sensory dorsal root ganglion (DRG) neurons requires close proximity (likely a synapse) between DRG neurons and neuropod cells (Barton et al., 2023). Further, as mentioned previously, Kaelberer et al. used rabies tracing to reveal that neuropod cells form direct synaptic connections with vagal nodose neurons (Kaelberer et al., 2018). Thus, the mediators of neuropod cell communication in this sense would function as a neuropeptide or a neurotransmitter, communicating synaptically.

Two neurotransmitters that have been associated previously with neuropod cells are glutamate and serotonin. CCK+ neuropod cells communicate with vagal nodose ganglion neurons through glutamate (Kaelberer et al., 2018; Buchanan et al., 2022). Co-culturing neuropod cells with HEK cells transfected with iGluSnFR (a reporter construct for glutamate) revealed GluSnFR activity in HEK cells after the neuropod cells were stimulated with glucose. Further, the glutamate receptor inhibitor kynurenic acid abolished vagal neuron responses to optogenetically stimulated neuropod cells, indicating that vagal neurons respond to neuropods with glutamate receptors (Kaelberer et al., 2018). When a cocktail of glutamate receptor inhibitors (Kynurenic acid, and AP3 for metabotropic glutamate receptors) was injected IP, mice could no longer respond to neuropod cell stimulation (Buchanan et al., 2022). Further, colorimetric assays of glucose in supernatants from organoids showed that glutamate was released from organoids after sucrose wash-in, but not after artificial sweeteners (Buchanan et al., 2022). This implicates glutamate as an essential mediator of the interaction between neuropods and the vagus nerve, specifically for nutrient sensing.

ECs are EECs characterized by their serotonin (5-HT) expression. Though not specifically described as neuropod cells in the literature, ECs have properties of neuropod cells: expressing transcripts encoding presynaptic proteins and interacting with peripheral neurons that express the ionotropic serotonin receptor 5HT_3_R (Bellono et al., 2017). Serotonin secreted from EC cells may act in an endocrine or synaptic fashion from neuropod cell to neuron, and it’s possible that these two modes of communication could inform neurons about separate modalities (e.g., pain versus motility). A wide variety of stimuli elicit 5-HT release from ECs, including mechanical stimuli, noxious stimuli, nutritive stimuli, and infection (Li et al., 2000; Nozawa et al., 2009; Hagbom et al., 2011; Alcaino et al., 2018). EC-containing organoids co-cultured with HEK cells transfected with 5HT_3_R were reactive to microbial metabolites and the noxious chemical allyl isothiocyanate (AITC) by releasing 5-HT and inducing calcium ion transients in HEK cells (Bellono et al., 2017). Washing AITC and microbial metabolites onto the intestinal lumen also induced firing in nociceptive nerve fibers in ex-vivo preparations (Bellono et al., 2017). In addition, a recent study implicated serotonin as a driver of visceral hypersensitivity and anxiety (Bayrer et al., 2023). Bayrer et al. showed that specifically silencing EC cells resulted in decreased colonic sensitivity to chemical and mechanical irritants (Bayrer et al., 2023). Activating EC cells led to an increase in serotonin release, increased visceral hypersensitivity, and produced anxiety behaviors (Bayrer et al., 2023). Each of these outcomes was abolished by a serotonin receptor antagonist (Bayrer et al., 2023). Together, these data suggest that serotonin released from neuropod cells modulates nociceptive neuron signaling, though this direct effect was not shown.

## 3. EECs receive signals from the intestinal lumen

To transduce luminal signals, EECs express specialized receptors for a host of different modalities: microbial metabolites (GPR40, short chain fatty acids), noxious stimuli (Trpa1, mustard oil and indole), stretch (Piezo2, mechanical), and even taste (TR2, bitter taste) (Nozawa et al., 2009; Janssen et al., 2011; Alcaino et al., 2018; Lu et al., 2018). The intracellular machinery that leads to hormone release after these receptors are stimulated have yet to be fully understood. Calcium influx is the canonical method for synapse release in neurons, and has also been shown to cause hormone release from EECs, and be inhibited by high fat diet (Ye et al., 2019). Cyclic nucleotide signaling, especially signaling through adenylate cyclase, is another positive regulator of hormone vesicle release (Friedlander et al., 2011). Forskolin, a cell permeable direct agonist of adenylate cyclase, is commonly used in epithelial preparations to induce hormone release from EECs, and has been used to measure the secretome from human organoids (Koop and Buchan, 1992; Beumer et al., 2020).

### 3.1. Nutrient sensing receptors

Because EECs interact with the intestinal lumen, they are often the first response to digestive products. EECs sense a variety of nutrients using transmembrane receptors that transduce luminal signals into intracellular signals. Sugar sensing has been a rich area of study in EECs, primarily because of the insulin-sensitizing hormones (incretins) secreted by EECs. One class of receptors that sense sugars are G-protein coupled taste receptors divided into two families: the T1Rs which taste sweet and umami, and the T2Rs which detect bitter taste (Latorre et al., 2016). The T1R family is comprised of three isoforms that function as heterodimers (Latorre et al., 2016). Of these heterodimers, T1R2-T1R3 has been identified as a receptor for intestinal glucose (Margolskee et al., 2007; Shirazi-Beechey et al., 2014). T1R2-T1R3 effects the sodium glucose transporter 1 (SGLT1), which is known to be regulated at the transcriptional and post-transcriptional level by luminal sugars (Dyer et al., 2003). A high carbohydrate diet increases SGLT1 mRNA and protein in EECs in wild type (WT), but not T1R3 knockout, mice showing that the taste receptor is necessary for downstream effects of sugar sensing (Margolskee et al., 2007). Interestingly, artificial sweeteners affected this pathway similarly, increasing SGLT1 expression in WT, but not T1R3 knockout, mice (Margolskee et al., 2007). This is notable because more recent studies have shown that the preference for sugar over artificial sweetener is mediated by neuropod cells, which signal synaptically to the vagus nerve in the lamina propria in the presence of sugars (Buchanan et al., 2022). Buchanan et al. showed that neuropod cells are enriched in transcripts for T1R3 and SGLT1 (Buchanan et al., 2022). When either T1R3 or SGLT1 was blocked by pharmacological agents, synaptic transmission to the vagus nerve was reduced following stimulation by sucrose but not the artificial sweetener sucralose (Buchanan et al., 2022). This is in contrast to previous studies, which showed no difference between sucrose and sucralose signaling through T1R3 (Margolskee et al., 2007). Reconciliation of these two opposing observations could help us understand the role artificial sweeteners play in the appetitive response. Further understanding of sugar-sensing mechanisms and the role of T1R3 in EECs also will facilitate the development of safe and effective treatments for diabetes and metabolic disease.

EECs also sense amino acids, activating distinct signaling pathways from sugar sensing (Symonds et al., 2015). A wide variety of amino acid receptors have been identified in EECs, due to the wide range of protein digestion products that are being sensed. Amino acids can be sensed by the T1R1-T1R3 heterodimer, which is primarily responsible for umami taste (as opposed to T1R2-T1R3, the sugar sensing heterodimer) (Latorre et al., 2016). The amino acid receptor GPRC6A also has been identified in EECs, though identification of its ligands and role in eliciting hormone release have been controversial (Steensels and Depoortere, 2018). Activation of GPRC6A leads to the release of GLP-1 from EECs *in vitro* (Oya et al., 2013). However, activation of GPRC6A with L-arginine and L-ornithine *in vivo* did not release GLP-1, binging into question the role of GPRC6A in amino acid sensing in EECs (Clemmensen et al., 2017). Another important amino acid receptor expressed by EECs is the calcium sensing receptor, CasR which has been linked to the release of several hormones including GLP-1, CCK, and PYY (Mace et al., 2012; Nakajima et al., 2012; Diakogiannaki et al., 2013). CasR also is upregulated in EECs expressing CCK, suggesting a unique ability of CCK-expressing cells to respond to specific amino acid products (Bai et al., 2022).

Additionally, EECs also can sense free fatty acids (FFAs). Medium and long chain FFA products of digestion are detected by FFAR1 and FFAR4 which are upregulated in EECs (Edfalk et al., 2008; Parker et al., 2009; Adriaenssens et al., 2019). The role of each receptor has not been fully defined. Eliminating *Ffar1* expression in mice reduced CCK, GIP, and GLP-1 secretion, while eliminating *Ffar4* expression impaired GIP secretion (Edfalk et al., 2008; Liou et al., 2011; Iwasaki et al., 2015). However, neither knockout completely abolished hormone secretion suggesting that the two receptors function in concert or that other pathways are involved. Short chain fatty acids (SFCAs) originate primarily from microbial fermentation, and are detected by FFAR2 and FFAR3. These receptors are concentrated in the large intestine, where most fermentation and SCFA production occurs (Steensels and Depoortere, 2018; Blaak et al., 2020). Fatty acids induce GLP-1 release from murine primary cultures of colonic cells, an effect diminished in FFAR2 and FFAR3 knockout mice (Tolhurst et al., 2012). SCFA sensing through FFAR receptors is an area of ongoing research because they may mediate a direct effect of the gut microbiome on hormone release from the intestine.

### 3.2. Microbiome sensing receptors

The human intestine harbors an estimated 38 trillion microorganisms, compared to the 30 trillion cells in the human body and meaning we are comprised of about 50% human cells and 50% microbial cells (Sender et al., 2016). Though microbiomes differ greatly from human to human, each individual’s healthy adult microbiome remains stable over time (Costello et al., 2009). A healthy gut microbiome confers many benefits to the human host including metabolism of food and drugs, protection from pathogenic microbes, maintenance of the gut mucosal barrier, immunomodulation, and likely other mutualistic effects that we have not yet discovered (Jandhyala et al., 2015). The homeostatic interplay between luminal gut cells and microbes is essential to maintaining a healthy microbiome and a healthy host. Dysregulation of the microbiome resulting in loss of beneficial microbes, loss of diversity, and/or excessive growth of harmful organisms leads to dysbiosis: intestinal bacterial disruption (DeGruttola et al., 2016). Dysbiosis caused by cytotoxic therapies like chemotherapy can be reversed by autologous fecal microbiota transfer, which can restore populations of beneficial microorganisms (Malard et al., 2021). Dysbiosis may contribute to an incredible array of diseases from GI syndromes like Crohn’s disease and ulcerative colitis, to Parkinson’s disease, hypertension, chronic kidney disease, hepatitis, asthma, and increased cancer risk (Hou et al., 2022). Thus, surveillance and communication of the state of the microbiome is an essential role for the intestine and EECs to maintain not only intestinal homeostasis, but the health of nearly every organ system.

To sense the microbiome EECs use a variety of receptors to detect microbial components, catabolites, and toxins. Toll-like receptors (TLRs) are pattern recognition receptors characterized by their ability to detect patterns exclusive to microbes (especially pathogenic microbes) (Medzhitov, 2001). These receptors were initially described as the mediator for innate immune cells detection of microbial components, and EECs have also been shown to express TLRs (Bogunovic et al., 2007; Palazzo et al., 2007; Lebrun et al., 2017; Daly et al., 2020). TLR4, the receptor for lipopolysaccharides (LPS), is expressed by EECs, and LPS increases both CCK and GLP-1 secretion in cell lines and mice (Bogunovic et al., 2007; Lebrun et al., 2017). TLR9, which detects unmethylated CpG islands (a differentiator of microbe DNA from vertebrate DNA), also is expressed by EECs. Similarly to TLR4, TLR9 activation increases CCK secretion (Daly et al., 2020). TLRs activate the NF-ΚB pathway in EECs, which directly increases transcription of hormones (e.g., PYY, whose promoter contains 2 potential NF-KB binding sites) (Bogunovic et al., 2007; Larraufie et al., 2017). In addition to directly sensing microbes, EECs can detect byproducts exclusively produced by the microbiome. Transient receptor potential ankyrin 1 (TRPA1) is expressed by EECs in mice, humans, and zebrafish, and detects microbial catabolites of tryptophan (e.g., indole) (Nozawa et al., 2009; Cho et al., 2014; Ye et al., 2021). In zebrafish, the bacteria *Edwardsiella tarda* catabolizes tryptophan to indole which acts on TRPA1, leading to increased motility by activating cholinergic enteric neurons and vagal pathways (Ye et al., 2021). In mammals, TRPA1+ EECs are located in the small intestine while *Edwardsiella tarda* is primarily in the colon (Nozawa et al., 2009; Cho et al., 2014). Thus, it’s possible that TRPA1+ EECs react to pathogenic and/or ectopic indole-producing microbes in the small intestine, increasing motility to attempt to rid indole-producing bacteria from the small intestine. EECs also can “smell” microbial metabolites through the olfactory receptor 558 (OLFR558), which detects isovalerate (Bellono et al., 2017). Activation of OLFR558 and TRPA1 in ECs increases firing of nociceptors, perhaps serving as a sensor for dysbiosis in the intestine (Bellono et al., 2017). The exact role of EEC activation and hormones in response to microbes has not been fully elucidated, and is likely highly complex, context-and hormone-dependent. In general, microbes seem to trigger heightened responses in EECs. Further, mice grown without microbes (germ-free mice) exhibit decreased levels of circulating serotonin (released by EC cells) (Hata et al., 2017). However, germ-free mice exhibit the opposite for GLP-1 and insulin-like peptide-5, having increased serum levels of both (Wichmann et al., 2013; Lee et al., 2016). The complexity of the microbiota and its effect on EECs is just beginning to be understood, and additional research may help us understand these opposing findings.

## 4. EECs are electrically excitable

Emerging evidence shows that a variety of EECs are electrically active and are capable of firing action potentials (APs) (Kaelberer et al., 2018). An AP is a coordinated event in which ion channels open and close in an orchestrated manner to transmit membrane voltage changes across the cell. To be capable of firing APs, cells must contain necessary voltage-gated ion channels. These typically include sodium and potassium channels—which serve the purpose of depolarizing and repolarizing the cell respectively. Furthermore, these cells must contain voltage-gated calcium channels—which are necessary for vesicular release of transmitters at the synaptic terminal. Together, the complete complement of ion channels in EECs allow these cells to rapidly respond to stimuli by secreting hormones and neuropeptides.

Previous studies revealed that L cells (GLP-1-predominant EECs) are electrically active (Rogers et al., 2011; Gribble and Reimann, 2016). Using molecular approaches, these studies described differential expression of voltage-gated ion channels – including sodium (Nav), potassium (Kv), and calcium (Cav) channels in Glutag cells and purified colonic L-cells (Rogers et al., 2011). Like pancreatic beta cells, glucose closes K(ATP) channels in L-cells allowing them to become excited and release hormone (Reimann and Gribble, 2002; Friedlander et al., 2011). In turn, the secreted GLP-1 (an incretin) induces pancreatic insulin release, a nutritive pathway that is disrupted in diabetes (Holst, 2007).

Additional studies of other EECs have shown that they too contain voltage-gated ion channels necessary for generation of APs (Bellono et al., 2017). Transcriptome sequencing of these EECs showed expression of Nav1.7, Kv7.1, as well as Cav3.2, and Cav2.1 (Bellono et al., 2017). It was also shown that these EECs are capable of firing APs on stimulation by irritants such as AITC. These cells are not only capable of firing APs, but in fact, EEC activation leads to voltage-gated calcium influx-mediated serotonin release (Bellono et al., 2017).

### 4.1. Sodium channels

A variety of different voltage-gated sodium channels (Navs) are involved in EEC signal transduction. Nav1.3, Nav1.7, and Nav1.8 (encoded by *SCN3A, SCN9A*, and *SCN10A* respectively), are expressed in EECs, and their voltage-dependent activation allows the influx of sodium ions, leading to the initiation of APs (Camilleri, 2013; Hughes et al., 2013; Strege et al., 2017). Generally, EECs are depolarized by chemical stimuli that trigger a depolarization of the EEC’s membrane potential causing Nav channel opening. The sodium channel Nav1.3 mediates spontaneous firing of EECs (Serotonergic, Tph1+) cells, and is necessary for these cells to fire APs (Strege et al., 2017). Disruptions in Nav1.3 function can impact hormone secretion, affecting various physiological processes such as nutrient absorption and gut motility, potentially contributing to GI disorders. Nav1.7 is an integral component of the molecular machinery underpinning the sensory functions of EECs. Nav1.7 is primarily expressed in peripheral sensory and sympathetic neurons (Dib-Hajj et al., 2007). However, emerging evidence suggests that Nav1.7 also plays a role in EECs (Rogers et al., 2011; Barton et al., 2023). Voltage-gated sodium channels require moderate cellular depolarization to activate and initiate the AP. Activation of non-specific cation channels like TRPA1 (previously mentioned as irritant receptors in EECs) can serve as an initial depolarizing step to open sodium channels like Nav1.7 (Bellono et al., 2017). EECs also express the mechanically-activated nonselective cation channel Piezo2 (Coste et al., 2010; Knutson et al., 2023). Thus, in addition to irritants, mechanical stimuli could also evoke APs in EECs.

Nav1.8 is a voltage-gated sodium channel found in sensory neurons. Similar to Nav1.7, Nav 1.8 has been extensively studied in the context of pain (Dib-Hajj et al., 2010). However, its role in EECs is not as clearly defined as that of Nav1.7. Nav1.8 is known for its unique property of activating at more negative potentials compared to other voltage-gated sodium channels, which may underlie a hyperexcitable state in EECs (Akopian et al., 1996; Xiao et al., 2019).

### 4.2. Potassium channels

Voltage-gated potassium channels (Kvs) are fundamental to the maintenance of cellular excitability, setting the resting membrane potential, and shaping APs and firing patterns in various cell types, including EECs. In EECs, Kvs contribute to the regulation of hormone secretion. Their activity influences the timing and amplitude of APs, thereby controlling the duration of voltage-gated calcium channel opening and the subsequent calcium influx, which is necessary for vesicular hormone release. EECs robustly express Kv7s and differentially express subtypes of Kv3s (Cosme et al., 2021; Barton et al., 2023).

EECs are enriched in Kv3 channels, including Kv3.1 (*KCNC1*) and Kv3.4 (*KCNC4*) (Rogers et al., 2011; Barton et al., 2023). Kv3 channels activate and deactivate rapidly at relatively positive membrane potentials. This gives cells that express Kv3 channels short refractory periods, and the ability to fire rapidly at rates from 100 to 600 Hz (Kaczmarek and Zhang, 2017). By doing so, Kvs play a role in regulating synaptic transmission (Ritter et al., 2015; Rowan and Christie, 2017; Muqeem et al., 2018). The role for these channels in EECs, and especially neuropod cells is likely multifactorial. Kv3.1 is expressed throughout the central auditory system, highlighting the importance of repetitive firing on transducing sensory stimuli (Li et al., 2001). Further, expression of Kv3.1 and rapidity of neuron firing is increased in neurons that respond to higher frequencies of sound (Brew and Forsythe, 2005). Kv3.1 expression and activity is therefore used to “tune” neurons to stimuli, increasing firing rates to encode qualitatively different signals. Thus, it is tempting to speculate that Kv3.1 expression is differential among EECs. It is possible that higher expression in some EECs favors rapid firing for tonic/sustained stimuli (e.g. pain), versus lower expression in EECs that receive nutritive signals. Recent description of the structure allows for rational drug design to target Kv3.1, presenting the opportunity to modulate repetitive firing and hormone and neuropeptide release from EECs (Chi et al., 2022). Kv3.4, on the other hand, is found in L-cells but is not found in neuropod cells (Rogers et al., 2011; Barton et al., 2023). It is possible that, in L cells, Kv3.1 and Kv3.4 play similar roles in AP repolarization and synaptic transmission modulation – but Kv3.4 is susceptible to protein-kinase C (PKC) modulation while the Kv3.1 is not. (Ritter et al., 2015; Alexander et al., 2022) Targeting PKC in L cells could therefore allow for pharmaceuticals that effect only GLP-1 secretion (through Kv3.4), but not neuropod dynamics.

Kv7 channels are expressed in many tissues including the heart and gut. EECs mostly express Kv7.1 (*KCNQ1*) and Kv7.2 (*KCNQ2*) (Jepps et al., 2021). Kv7s exhibit slow kinetics and low voltage activation and are responsible for regulating repetitive firing of neurons (Dedek and Waldegger, 2001; Demolombe et al., 2001). In the context of EECs, Kv7.1 plays a critical role in potassium secretion and recycling. This process is essential for maintaining the electrical driving force for electrolyte secretion, particularly in EECs. The activation of Kv7.1 channels permits efflux of potassium ions from the cytosol to the extracellular space (Kunzelmann et al., 2001). Kv7.1 is predominantly located in the basolateral membrane of these cells, facing the bloodstream, and mediates potassium efflux from cells into the circulation, which helps return the membrane potential to the resting state following an AP. Association of Kv7.1 with KCNE1 produces channels that activate slowly with a depolarizing voltage and have a unique sensitivity to chromanol 293B, a specific KCNQ1 blocker (Lerche et al., 2007). This combination is particularly relevant in cardiac tissue, where it contributes to the repolarization phase of the cardiac AP. On the other hand, when Kv7.1 associates with KCNE3, it produces channels that activate almost instantaneously with depolarization and exhibit constitutive (voltage-independent) activity (Alzamora et al., 2011). This characteristic is important in colonic crypt cells, where Kv7.1/KCNE3 channels contribute to the resting membrane potential and regulate the driving force for chloride efflux, a crucial component of fluid secretion in the colon (Dedek and Waldegger, 2001; Alzamora et al., 2011). Further, other Kv7s, including Kv7.2 and Kv7.4 appear to be involved in K+ secretion (Inagaki et al., 2019).

### 4.3. Calcium-activated potassium channels

Calcium-activated potassium channels (KCa) have diverse roles in EECs, and their expression and location depend on the species and cell type. KCa1.1 (*KCNMA1*) channels, for example, are expressed in the basolateral membrane of rabbit colonocytes, while in rat and mouse colonocytes, these ion channels are predominantly found in the apical membrane, especially in crypts (Bowley et al., 2003). Interestingly, high potassium intake increases apical KCa1.1 channels, which may contribute to inflammatory bowel disease (IBD)-related diarrhea (Sausbier et al., 2006). In human colon, KCa1.1 channels are more specialized in location and function, expressed in apical membranes of goblet cells where they mediate potassium secretion (Singh et al., 2012). However, KCa1.1 channels are found along the entire crypt axis in patients with ulcerative colitis (UC).

KCa3.1 (*KCNN4*) channels are the primary basolateral potassium channels in human colonocytes, and they are distributed along the surface-crypt axis (Singh et al., 2012). The role of these channels in intestinal pathophysiology is complex. Although some studies suggest they are downregulated in UC and Crohn’s disease, others have found higher levels of KCa3.1 mRNA in the intestinal epithelium of IBD patients (Barmeyer et al., 2010; Basalingappa et al., 2011). These discrepancies might be related to disease activity, with elevated KCa3.1 levels potentially promoting wound healing. Interestingly, clotrimazole inhibition of these ion channels in EECs enhanced wound healing after mechanical injury, implying that potassium channel modulation may regulate cell migration in different ways under inflammatory and non-inflammatory conditions.

### 4.4. Role of Ca channels in vesicle release from EECs

Calcium channels play an essential role in neurotransmission (Dolphin and Lee, 2020). Previous studies revealed that neuropeptides are more strongly released in response to voltage-gated calcium channel activation in sensory neurons compared to ligand-gated calcium channel activation (e.g., via TRPV1) (King et al., 1999; Wang et al., 2017). Voltage-gated calcium channels (VGCCs) represent a key class of ion channels implicated in vesicle release in EECs (Catterall, 2011; Zamponi et al., 2015). In neurons, voltage gated calcium channel activation and calcium influx induces an orchestrated binding of vesicles and release of neurotransmitter (Gustavsson et al., 2011; Bohórquez et al., 2015; Bellono et al., 2017). In EECs, activation of P/Q type voltage-gated calcium channels led to the release of serotonin (Bellono et al., 2017). Interestingly, activation of TRPA1 and TRPC4 calcium channels—which could cause large increases in intracellular calcium—were not sufficient to cause neurotransmitter release. Likely this is due to their location within the cell relative to the release sites. Furthermore, blockage of voltage-gated presynaptic calcium channels by ω-agatoxin IVA was sufficient to inhibit depolarization-evoked responses (Bellono et al., 2017). Therefore, voltage-gated calcium channels may play a key role in the release of hormones from EECs.

## 5. Role of EECs in human disease

EECs are essential regulators of homeostasis, evidenced by syndromes in which their elimination produces devastating disease. Neurogenin-3 (*NEUROG3*), is a transcription factor essential for the differentiation of EECs in the intestine, as well as endocrine cells of the pancreas, and hypothalamus (Gradwohl et al., 2000; Jenny et al., 2002; Pelling et al., 2011; Spence et al., 2011). NEUROG3 knockout mice have helped to elucidate the essential role of both NEUROG3 and EECs in homeostasis. Notably, elimination of NEUROG3 in mouse intestine leads to intestinal malabsorption and 50% lethality within the first 8 days of life (Mellitzer et al., 2010). Interestingly, intestinal malabsorption and diarrhea in mice without intestinal EECs can be partially rescued by intraperitoneal PYY administration (McCauley et al., 2020). Thus hormone-replacement therapy may serve as a possible solution to syndromes related to EEC loss.

### 5.1. Neurogenin 3 mutations

In humans, mutations in NEUROG3 lead to dysgenesis of EECs in the intestine and pancreas and subsequent life-threatening malabsorption and diabetes, a syndrome termed “enteric anendocrinosis” (Wang et al., 2006). Patients with NEUROG3 mutations exhibit almost complete loss of EECs throughout the small intestine and colon, and what few EECs remain in these intestines express much less chromogranin A (marker of EECs) (Cortina et al., 2007). As of 2023, 17 cases of pathologic NEUROG3 variation have been reported, all with malabsorptive diarrhea (Wejaphikul et al., 2023). Universally, these patients require parenteral nutrition, though the exact mechanism of intestinal malabsorption in the absence of EECs is not known. NEUROG3 canonically induces differentiation by promoting transcription of NEUROD1, and the reported NEUROG3 mutations produce loss of this function (Huang et al., 2000). Interestingly, NEUROD1 mutations do not lead to EEC dysgenesis and enteric anendocrinosis, but have been correlated with cases of type 2 diabetes mellitus (Malecki et al., 1999; Brodosi et al., 2021). Absence of EECs at birth results in an intestine unable to perform its primary role: nutrient absorption. Thus, enteroendocrine dysgenesis reveals the essential nature of EECs to the intestine, and to overall survival in newborns. A recent study eliminated EECs from adult mice by crossing Neurog3^flox/flox^ mice with the villin-CreER^T2^ line and administering tamoxifen in 8–13 week old males (Blot et al., 2023). After EEC elimination, males fed standard chow diet lost 30% of their fat mass and experienced significant weight loss compared to mice with healthy EECs. Males without EECs fed high fat diet, however, fared much worse, and 60% died 5 weeks after tamoxifen administration, and the remainder were euthanized for profound malnutrition (Blot et al., 2023). It is unclear if EEC loss has the same effect in female mice, or why they were excluded from these experiments. However, this study revealed that EEC loss is not universally lethal in adults, though it does significantly impair intestinal absorption, especially lipid absorption.

### 5.2. ARX and PCSK1 mutations

Enteric anendocrinosis is also seen in patients with mutations in the ARX (Aristaless-Related Homeobox) gene (Kato and Dobyns, 2004; Terry et al., 2015; Coman et al., 2017). Unlike NEUROG3 loss of function mutations, ARX loss of function mutations do not completely eliminate EECs. Instead, ARX mutations shift the profile of EECs, while maintaining total EEC number. CCK-and GLP-1-producing EECs are most profoundly affected, with near-complete elimination in patients with ARX variants, while overall chromogranin A (ChgA)-producing cells in the intestine are maintained (Collombat et al., 2003; Terry et al., 2015). Similarly, deficiency of proprotein convertase subtilisin/kexin type 1 (PCSK1) gene products also results in congenital malabsorptive diarrhea (Bandsma et al., 2013; Ahmed and Alsaleem, 2021; Lotta and Ferreira, 2021; Ni et al., 2022). However, patients with PCSK1 variants recover and wean off of parenteral nutrition after infancy, unlike those affected by ARX and NEUROG3 loss of function. Similar to ARX mutations, PCSK1 mutations do not affect the overall number of ChgA-expressing cells in the duodenum, but result in a decrease in GLP-1-expressing cells (CCK was not tested) (Bandsma et al., 2013). These phenotypes bear striking similarity to intestinal Islet1 (Isl1) knockout mice, which exhibit an unchanged overall ChgA cell population, but a dramatic loss of GIP, GLP-1, CCK, and Sst cells in favor of an increase in 5-HT producing cells (Terry et al., 2014). Intestinal Isl1 knockout mice also experience malabsorptive diarrhea and impaired glucose tolerance (Terry et al., 2014). Together, ARX, PCSK1, and Isl1 mutations emphasize the necessary balance of EEC subtypes and hormones necessary to maintain homeostasis and allow for proper gut function. Though EECs are still produced in individuals with these mutations, even small shifts in EEC hormone production can have profound effects on intestinal function.

### 5.3. Autoimmune destruction of EECs

Mutations extrinsic to EECs also cause EEC destruction and dysfunction. Autoimmune regulatory (AIRE) gene is primarily expressed by medullary cells in the thymus, which “educate” T cells to determine antigens that are “self” versus “non-self” (Gardner et al., 2009). Loss of function mutations in AIRE result in autoimmune polyendocrinopathy-candidiasis-ectodermal dystrophy (APECED) also known as Autoimmune Polyglandular Syndrome type 1 (APS-1). This syndrome is characterized by a triad of chronic mucocutaneous candidiasis, hypoparathyroidism, and Addison’s disease (Meloni et al., 2012). Clinical presentation of this syndrome is heterogeneous and often includes additional features, such as gonadal failure, diabetes mellitus, and intestinal malabsorption in 25% of patients (Ahonen et al., 1990; Gianani and Eisenbarth, 2003; Sköldberg et al., 2003). In fact, 10% of patients with APS-1 initially present with GI symptoms (Kluger et al., 2013). While intestinal malabsorption was originally attributed as non-endocrine autoreactivity in APS-1, further investigation revealed loss of EEC subtypes in the stomach (enterochromaffin-like cells) and intestine (CCK-producing cells) (Högenauer et al., 2001; Sköldberg et al., 2003). Further, antibodies to tryptophan hydroxylase (the rate-limiting enzyme in serotonin synthesis and a primary marker for ECs) were detected in one third of patients with APS-1 (Ekwall et al., 1998). These findings highlight EECs in the intestine as an endocrine “unit.” Though EECs are not contiguous like the endocrine cells of the adrenal gland, they are similarly susceptible to autoimmune attack of endocrine organs. The ability for EECs to be selectively attacked by the immune system also implicates immunomodulation as a possible method of altering the balance of EEC subtypes in the intestine.

### 5.4. EECs and irritable bowel syndrome

Investigation of EECs in patients with IBS reveals diverging roles of individual hormones between different subtypes of IBS. IBS patients express fewer (71% decrease) duodenal Neurog3+ cells compared to healthy patients (an average of 103 in IBS versus 351 in healthy) (El-Salhy et al., 2015). Using ChgA as an EEC marker, a study of 25 patients with diarrhea-predominant IBS (IBS-D) found no difference in EEC number in the ileum, colon, or rectum compared to healthy patients (Park et al., 2006). However, a study with greater power (101 patients) found significantly reduced total ChrgA+ cells in the ileum in patients across all types of IBS (El-Salhy and Gilja, 2017). This study also found significantly reduced cell Neurogenin3 and serotonin –expressing cells across all types of IBS, as well as decreased Msi-1 cells (general stem cell marker). Most interestingly, the number of cells producing PYY were *increased* in IBS-C patients only, with an upward trend in mixed IBS (IBS-M) (El-Salhy and Gilja, 2017). However, in rectal samples, the opposite trend was observed in which patients with all subtypes of IBS exhibit decreased PYY cells (El-Salhy et al., 2014). In the duodenum, CCK, GIP, and somatostatin –expressing cells were reduced in all IBS subtypes (El-Salhy et al., 2015). Ghrelin, like PYY, differs between IBS subtypes, with an increase in ghrelin-expressing cells in IBS-D and a decrease in IBS-C (El-Salhy et al., 2009). The ghrelin study stands out as the only one in which serum levels of hormone were assessed, and though cell numbers differed, serum levels of ghrelin were not different in IBS patients. Studies on IBS patients after fecal transplant and after dietary guidance to alleviate IBS symptoms all revealed that IBS symptoms correlate with a decrease in EEC number, while IBS symptom relief is associated with an increase in EEC number (Mazzawi and El-Salhy, 2016; Mazzawi et al., 2022). Notably, all studies observing significant differences in EEC numbers between IBS patients and control patients were performed by the same investigators, while the one study revealing no difference in EEC numbers was from a different laboratory (El-Salhy et al., 2009, 2014, 2015; Mazzawi and El-Salhy, 2016; El-Salhy and Gilja, 2017; Mazzawi et al., 2022). Assays of fecal chromagranins and secretogranins (products of EECs), revealed increased ChrgA, Sct2, and Sct3, but decreases in ChrgB, in adult IBS patients (Öhman et al., 2012). Similarly, fecal ChrgA and Sct3 were greater in children with IBS (Shulman et al., 2019). ECs also are upregulated in post-infectious IBS, and serotonin levels are increased in rectum samples from IBS-C patients, supporting the fecal chromogranin data (Table 2; Miwa et al., 2001; Qin et al., 2019). Together, these data reveal that most EEC types in the duodenum are decreased in IBS. In the distal intestine, the consensus on the fate of EECs in IBS is much less clear. Neuropod cells are enriched in the duodenum, rather than the distal intestine, meaning that altering these cells in the duodenum could contribute to the etiology of IBS (Barton et al., 2023).

**Table 2:** Enteroendocrine changes in IBS patients.

Marker	Type of IBS studied	Change with IBS	Sampling site	Citation
ChrgA	IBS-D	No difference in cell number Negative correlation with pain threshold	Ileum, Colon, Rectum	Park et al. (2006)
ChrgA	IBS-C, -M, -D	Decrease in cell number	Ileum	El-Salhy and Gilja (2017)
Neurog3	IBS-C, -M, -D	Decrease in cell number	Duodenum	El-Salhy et al. (2015)
Neurog3	IBS-C, -M, -D	Decrease in cell number	Ileum	El-Salhy and Gilja (2017)
Serotonin	IBS-C, -M, -D	Decrease in cell number	Ileum	El-Salhy and Gilja (2017)
Serotonin	IBS (unspecified)	Decrease in cell number	Duodenum	Mazzawi and El-Salhy (2017)
Serotonin	IBS-C, -M, -D	No change in cell number	Duodenum	El-Salhy et al. (2015)
Serotonin	IBS-C	No change in serum levels	Plasma	Atkinson et al. (2006)
Serotonin	IBS-D	Increased serum levels	Plasma	Atkinson et al. (2006)
Serotonin	IBS-C	Increased tissue levels	Rectum	Miwa et al. (2001)
Serotonin	IBS-D	No change in tissue levels	Rectum	Miwa et al. (2001)
PYY	IBS-C	Increase in cell number	Ileum	El-Salhy and Gilja (2017)
PYY	IBS-D	No change in cell number	Ileum	El-Salhy and Gilja (2017)
PYY	IBS-C, -M, -D	Decrease in cell number	Rectum	El-Salhy et al. (2014)
CCK	IBS-C, -M, -D	Decrease in cell number	Duodenum	El-Salhy et al. (2015)
CCK	IBS (unspecified)	Decrease in cell number	Duodenum	Mazzawi and El-Salhy (2017)
GIP	IBS-C, -M, -D	Decrease in cell number	Duodenum	El-Salhy et al. (2015)
GIP	IBS (unspecified)	Decrease in cell number	Duodenum	Mazzawi and El-Salhy (2017)
Somatostatin	IBS-C, -M, -D	Decrease in cell number	Duodenum	El-Salhy et al. (2015)
Somatostatin	IBS (unspecified)	Decrease in cell number	Duodenum	Mazzawi and El-Salhy (2017)
Secretin	IBS-C, -M, -D	Decrease in cell number	Duodenum	El-Salhy et al. (2015)
Secretin	IBS (unspecified)	Decrease in cell number	Duodenum	Mazzawi and El-Salhy (2017)
Ghrelin	IBS-C	Decrease in cell number No change in tissue or serum levels	Duodenum	El-Salhy et al. (2009)
Ghrelin	IBS-D	Increase in cell number No change in tissue or serum levels	Duodenum	El-Salhy et al. (2009)

### 5.5. EECs and obesity

EECs’ role in metabolism and appetite regulation is the subject of many comprehensive reviews, the full scope of which we cannot cover here (Symonds et al., 2015; Ricardo-Silgado et al., 2021; Gribble and Reimann, 2022). However, the change in EEC hormones after bariatric surgery is intriguing and sheds a light on the dysregulation of EECs in human obesity. These bariatric surgery studies provide an essential paired case study comparing patients before and after weight-loss surgery. Individuals with obesity have decreased overall levels of GLP-1-expressing EECs, a deficit that is more pronounced in individuals with obesity and type 2 diabetes (Osinski et al., 2021). A comprehensive study of duodenal tissue from patients with and without obesity revealed a decrease in overall EEC number and a decrease in most EEC hormones in obesity (Wölnerhanssen et al., 2017). After sleeve gastrectomy, EEC cells and EEC hormones in patients with obesity increased to levels similar to patients without obesity. Other studies have found similar results, with EEC numbers increasing after sleeve gastrectomy and roux en y gastric bypass (Peterli et al., 2012). These findings also are conserved across species, as mice and rats that undergo bariatric surgery also show significant upregulation of EECs and circulating EEC hormones (Nausheen et al., 2013; Li et al., 2016).

Though decreased EECs and EEC hormones are correlated with obesity, it is unclear whether this is cause or effect. Fascinating experiments in zebrafish showed that EECs are less reactive to nutrients in fish maintained on a high fat diet (HFD) (Ye et al., 2019). Early timepoints in mice started on HFD showed moderate decreases in EECs and a shift from secretory lineages in the intestine to enterocyte lineages (Enriquez et al., 2022). Similarly, after 12 weeks of HFD mice show a large shift within the secretory lineage, away from hormone-secreting EECs (decreasing all EEC types) and toward mucin-secreting goblet cells (Aliluev et al., 2021). The shift within secretory lineages may be ascribed to NEUROG3 level, as intestinal cells with high expression of NEUROG3 are fated to become EECs, while lower expression of NEUROG3 drives cells to become goblet cells (Li et al., 2021). These studies support the concept that high fat diet reduces EEC number and function. However, substantial evidence also exists for decreased EEC function leading to obesity. Notably, polymorphisms in EEC hormones and hormone receptors have been linked to obesity. One prominent example is the CCK receptor (*CCKAR*), polymorphisms which are associated with hyperphagia and obesity (Marchal-Victorion et al., 2002; de Krom et al., 2007). Similarly, polymorphisms in GLP-1 receptors (*GLP1R*) either increase (AA carriers of rs692376) or decrease (TT carriers of rs2268641) the risk of being overweight (Michałowska et al., 2022). Thus, this complex question has yet to yield a concrete answer as degradation of gut-brain signals can both be caused by, and cause, obesity. Likely, because obesity is a highly prevalent and heterogenous disease, diminished EEC signaling is both a driver and a consequence of obesity depending on the individual. Excellent reviews on the topic expand on this discussion (Lean and Malkova, 2016; Steinert et al., 2017).

### 5.6. EECs and functional dyspepsia

Functional dyspepsia (FD) is divided by the Rome IV criteria into two subtypes: postprandial distress syndrome (PDS) with postprandial fullness or early satiation, and epigastric pain syndrome (EPS) with epigastric pain or epigastric burning (Stanghellini et al., 2016). Much like IBS, FD is a diagnosis of exclusion, defined by a lack of structural disease, and with unclear pathogenesis (Wauters et al., 2020). Enteroendocrine hormone imbalance may play a role in the pathophysiology of FD. In a small study of 8 subjects with FD and 8 control subjects, postprandial plasma CCK was decreased and PYY was increased in subjects with FD (Pilichiewicz et al., 2008). Interestingly, ghrelin decreases sensitivity of vagal afferents in the upper GI tract, implicating ghrelin as a hormone that could modulate symptoms of FD (Page et al., 2007). However, studies investigating ghrelin plasma levels in FD have reached conflicting conclusions, with some studies finding decreased ghrelin in FD patients, and others finding no difference between patients with and without FD (Shindo et al., 2009; Kim et al., 2012). An excellent systematic review of GI hormones in FD was published recently and concludes that available studies are lacking in sample size and timing after standard meals (Van den Houte et al., 2020). More robust studies of FD populations (especially with respect to the two differing subtypes) could direct therapeutic management for this prevalent population.

## 6. Pharmacologic regulation of the gut brain axis

Gut hormones form the basis for many pharmaceuticals. Recently, they have come into the spotlight after the recent success of GLP-1 receptor agonists, glucagon analogues, and GIP analogues (Tschöp et al., 2023). Here, we describe two classes of drugs based on gut hormones, including agonists for GLP-1 and guanylyl cyclase C receptors.

### 6.1. Glucagon like peptide 1 receptor agonists

Semaglutide (Ozempic, Wagovy), is a GLP-1 receptor (GLP-1R) agonist that has garnered widespread attention recently for its role in appetite suppression and weight loss. New prescriptions for Novo Norodisk’s formulations of semaglutide Ozempic and Wegovy have experienced substantial growth, with new prescriptions of Wegovy increasing 297% in 2022 (Gilbert, 2023). The “Ozempic craze” has caused shortages of semaglutide formulations, especially effecting individuals with diabetes who use this agent primarily for its incretin effects (Food and Drug Administration, 2023; Smith, 2023). A wide body of literature supports the effects of semaglutide and long acting GLP-1R agonists on weight reduction, notably the STEP trails, which assessed the efficacy of once weekly 2.4 mg semaglutide injections to induce weight loss when coupled with lifestyle intervention (Ahrén et al., 2018; Yeh et al., 2023). In the STEP1 trial, individuals in the semaglutide group lost 14.9% of their original body weight compared to the 2.4% in the placebo group, resulting in a treatment difference of −12.4% body weight (95% CI −13.4 to −11.5) after 68 weeks (Wilding et al., 2021). The robust weight loss in the STEP1 trial exceeded the effects of liraglutide, another GLP-1R agonist, which caused a weight loss of only 5.4% in the SCALE trial (after 56 weeks of 3 mg per day). These superior weight loss results were recapitulated in a head to head comparison in the STEP 8 trial (Pi-Sunyer et al., 2015; Rubino et al., 2022). Significant weight loss also was observed in a cohort study in the clinical setting where overweight patients lost an average of 10.9% of starting body weight by 6 months (Ghusn et al., 2022). However, the STEP4 trial demonstrated that weight loss from semaglutide is not sustained, and study participants who were switched to placebo after 20 weeks regained 6.9% of their body weight by 68 weeks (Rubino et al., 2021). The SURMOUNT trial revealed that tirzepatide (Mounjaro, Eli Lily), an agonist of both GLP-1R and GIP receptors (GIPR) also significantly impacted weight loss in a dose dependent manner. Tirzepatide reduced initial weight by 15% (5 mg), 19.5% (10 mg), and 20.9% (15 mg), compared to 3.1% in the placebo group, after 72 weekly injections (Jastreboff et al., 2022). Though tirzepatide and semaglutide have not been directly compared in a clinical trial for weight loss, comparison of the STEP 1 and SURMOUNT-1 trials reveals an estimated cost needed to treat per 1% body weight reduction of $985 for tirzepatide ($17,527 for 72 weeks), versus $1845 for semaglutide ($22,878 for 68 weeks) (Azuri et al., 2023). Thus both options come at significant cost for weight reduction, though preliminary comparison shows enhanced value for tirzepatide compared semaglutide. Adverse events for these two expensive drugs also are not insignificant, with 19.2% of participants experiencing nausea and 13.7% experiencing diarrhea in a trial comparing tirzepatide and semaglutide effectiveness in type 2 diabetes (Frías et al., 2021). In a 2 year study of semaglutide injections, over half of the participants reported nausea (53.3%) and 82.2% experienced negative GI symptoms (compared to 53.3% of placebo) (Garvey et al., 2022). These results are not unique to semaglutide and tirzepatide, as nausea and diarrhea are the most common adverse effects of the GLP-1R agonists regardless of formulation (Trujillo, 2020).

The mechanism by which GLP-1R agonists induce weight loss has been well-studied, but has proven difficult to elucidate. GLP-1R is expressed both throughout the brain (amygdala, hypothalamus, ventrolateral medulla, nucleus of the solitary tract, thalamic paraventricular nucleus, hippocampus and cortex), and in the periphery by vagal and enteric neurons (Jensen et al., 2018). Similarly, GLP-1 is expressed not only in enteroendocrine L-cells, but also in preproglucagon-positive (PPG+) neurons in the nucleus tractus solitarius (Trapp and Cork, 2015). Untangling the effect of peripheral administration of GLP-1R agonists on peripheral receptors versus central receptors in the brain has been essential to defining how these drugs exert their anorexigenic action and how to minimize unwanted adverse effects. The question remains whether nausea induced by GLP-1R agonists is an off-target or on-target effect; i.e., whether nausea is coupled with hypophagia, or a sensation targeting receptors separate from appetitive pathways.

A key detail in determining how GLP-1R agonists act is the drugs’ ability to penetrate the blood brain barrier when administered peripherally. Some studies report that GLP-1R agonists can only act on circumventricular organs not protected by the blood brain barrier (Ørskov et al., 1996; Secher et al., 2014). However, other studies have shown fluorescently labeled GLP-1R agonists in the nucleus accumbens (a deep brain area) (Aranäs et al., 2023). Importantly, the action of PPG+ neurons on deep structures within the brain are not mimicked by administration of GLP-1R agonists (Brierley et al., 2021). This implies that the administration of GLP-1R agonists does not recruit the central PPG+ neuron pathway, but acts more similarly to L-cell released GLP-1. In support of this idea, peripheral administration of GLP-1R agonists directly bind and induce activation of POMC/CART anorexic neurons, and indirectly inhibit pro-feeding NPY/AgRP neurons in the arcuate nucleus (a circumventricular area) (Secher et al., 2014; Barreto-Vianna et al., 2016). Thus, GLP-1R agonists act like GLP-1 released from L cells, and unlike GLP-1 released from PPG+ neurons within the brain. Further, PPG+ neurons do not express GLP-1R, and their action is unaffected by peripheral administration of GLP-1R agonists (Trapp and Brierley, 2022).

Elegant studies inhibited central action of GLP-1R agonists by conjugating GLP-1R agonists to vitamin B12. These “corrinated” agonists could bind to GLP-1R outside of the brain, but not in the hypothalamus (responsible for feeding control) or area postrema (responsible for nausea) (Mietlicki-Baase et al., 2018; Borner et al., 2020). Using musk shrews (which, unlike mice, can vomit), these experiments revealed that central activation by GLP-1R agonists was necessary to induce vomiting and weight loss, but not glycemic control (Borner et al., 2020). GLP-1R agonists may induce nausea by binding to the area postrema activating glutamatergic neurons, or by action on the central amygdala, but data has differed based on species selection in studies (Lachey et al., 2005; Adams et al., 2018).

Though much of the research on GLP-1R agonists has focused on the brain and pancreas, GLP-1R also is expressed elsewhere in the body and on EECs themselves (Grunddal et al., 2022). In fact, GLP-1R is the most highly expressed peptide hormone receptor on ECs in mouse instestine (Lund et al., 2018). Serotonin release from ECs modestly increases following stimulation with liraglutide, a GLP-1R agonist (Lund et al., 2018). GLP-1R agonists also are powerful inducers of intestinal growth, and 1 week of GLP-1R agonist administration increases mouse intestinal length, weight, and circumference by increasing crypt number (Koehler et al., 2015). Thus, while GLP-1R agonists exert effects on peripheral and central neurons, their effects on the intestine itself are significant. Further investigation to elucidate the mechanisms by which GLP-1R agonists effect intestinal homeostasis and EEC function is important both to limit the GI side effects of these drugs and to enhance their long-term efficacy.

### 6.2. Guanylyl cyclase C agonists

As mentioned previously, guanylyl cyclase C (GUCY2C) is a transmembrane receptor expressed throughout the intestinal epithelium (Waldman and Camilleri, 2018). GUCY2C was first discovered for its role in intestinal secretion, as the receptor responsible for travelers’ diarrhea and the molecular target for the heat-stable enterotoxin (STa) produced by enterotoxigenic *E. coli* (ETEC) (Schulz et al., 1990, 1997). Beyond its role in intestinal secretion, GUCY2C also regulates intestinal enterocyte homeostasis (Rappaport and Waldman, 2018). In contrast to GLP-1R, GUCY2C activation inhibits cell growth. This property makes GUCY2C activation an attractive target as an antineoplastic (Entezari et al., 2021). More recently, GUCY2C also has been discovered in the brain, comprising neural circuits from the hypothalamus and midbrain (Merlino et al., 2019). Additionally, though GUCY2C is expressed throughout the intestinal epithelium, EECs express significantly increased levels of GUCY2C compared to enterocytes (Barton et al., 2023). Though the intracellular sequelae of GUCY2C activation in EECs have not been studied beyond cGMP production, GUCY2C activity in EECs decreases visceral pain in rodents and humans (Castro et al., 2013; Boulete et al., 2018; Barton et al., 2023). Further, GUCY2C agonism increases hormone release from GLP-1 cells and modulates communications between neuropod cells and neurons, implying that this receptor plays a role in regulating EEC signaling (Friedlander et al., 2011). Together, these recent discoveries make GUCY2C an attractive therapeutic target to modulate the gut-brain axis.

In healthy intestine, two endogenous luminal hormones regulate GUCY2C activity: guanylin (GUCA2A, secreted from the large intestine) and uroguanylin (GUCA2B, secreted from the small intestine) (Currie et al., 1992; Hamra et al., 1993). These two hormone ligands were first isolated from rat jejunum and kidney, and opossum urine, respectively (Currie et al., 1992; Hamra et al., 1993). Both hormones are secreted into the intestinal lumen and the systemic circulation (Hess et al., 1995; Date et al., 1996). Although these two hormones are structurally very similar, uroguanylin contains two N-terminal aspartic acid residues that support ligand-receptor affinity in acidic environments of in the small intestine (Hamra et al., 1997). Numerous groups have reported on subpopulations of epithelial cells producing guanylin and uroguanylin hormones. In both rat and human, guanylin protein has been identified in goblet cells of the differentiated cell compartment in small and large intestine (Cohen et al., 1995; Li et al., 1995; Brenna et al., 2016). Guanylin has also been found in surface epithelial cells of the colon and Paneth cells of small intestine (Cohen et al., 1995; Brenna et al., 2016). Conversely, the expression pattern of uroguanylin is not clearly defined. Early investigations identified uroguanylin expression in epithelial cells of rat and human duodenum, including cells with tuft-like morphology (Brenna et al., 2016). The expression pattern of guanylin and uroguanylin in EECs is highly debated in the literature. In ECs, some reports identify the absence of both ligands (Date et al., 1996; Brenna et al., 2016), while other reports identified expression in small intestinal ECs in rat, *Mastomys*, guinea pig, and human (Cetin et al., 1994; Hess et al., 1995; Hill et al., 1995; Perkins et al., 1997; Kidd et al., 2006). Most recently, transcriptomic analysis revealed downregulated expression of guanylin and uroguanylin in neuropod cells purified from mouse small intestine, compared to bulk intestinal epithelium (Barton et al., 2023). The discrepancies in cell-specific GUCY2C ligand expression may be species-dependent, as mouse studies differ from human, rat, *Mastomys*, and guinea pig. Thus, the cell-specific expression of guanylin and uroguanylin hormones remains insufficiently elucidated.

The role of ligand-activated GUCY2C in hormone regulation is a current topic of study. GUCY2C-deficient mice demonstrate a propensity for both hyperphagia and obesity (Valentino et al., 2011). Also, food intake in wild type mice triggers the release of prouroguanylin from the intestine into the systemic circulation. Subsequently, prouroguanylin reaches the hypothalamus and is proteolytic cleaved into the mature uroguanylin peptide. The presence of uroguanylin then stimulates GUCY2C activation and downstream appetite suppression pathways (Kim et al., 2013). Moreover, mice reduced food intake following intravenous administration of prouroguanylin and that effect was rescued following administration of prouroguanylin neutralizing antiserum (Kim et al., 2016). Interestingly, these studies were not mimicked with proguanylin, suggesting a uroguanylin-specific response (Valentino et al., 2011). Similarly, plasma concentrations of prouroguanylin, but not proguanylin, were decreased in obese individuals, and roux-en-Y gastric bypass-associated weight loss increased plasma proguanylin and prouroguanylin (Rodríguez et al., 2016). Additionally, mice eating a high-fat diet exhibited reduced intestinal guanylin expression, while re-expressing guanylin reduced the incidence of obesity-related colorectal cancer in these mice (Lin et al., 2016). Further, guanylin and uroguanylin mRNA expression was upregulated in chromogranin A-positive cells of the intestine following Roux-en-Y gastric bypass, similar to the increase seen with other gut hormones after bariatric surgery (Fernandez-Cachon et al., 2018). However, more recent studies failed to show that peripheral administration of GUCY2C ligands or prohormones alter food intake or glucose homeostasis in mice (Fernandez-Cachon et al., 2018). Moreover, central administration of guanylin and uroguanylin in some studies also did not affect food intake in rats (Begg et al., 2014; Fernandez-Cachon et al., 2018). Together, these conflicting data highlight the need for more studies to elucidate the effect of GUCY2C signaling on hormone regulation.

Guanylin and uroguanylin have striking homology to STa (the exogenous toxin produced by *E. coli*), making STa a textbook case of molecular mimicry (Ozaki et al., 1991; Fasano, 2002; Taxt et al., 2010). Compared to these endogenous ligands, STa has nearly a 10-fold higher affinity for GUCY2C, earning it the title of “superagonist” (Hamra et al., 1997). Because of its potent ability to induce secretion in the intestine, STa served as a blueprint for medications to treat constipation. Linaclotide, initially named MD-1100 acetate, is a 14 residue peptide analog of STa developed to potently activate GUCY2C (Bueno et al., 2004). Linaclotide was initially approved in 2012 to treat IBS with constipation (IBS-C) and chronic idiopathic constipation (CIC) under the brand name Linzess (Bryant et al., 2010; Thomas and Allmond, 2013). In 2017 a uroguanylin analog, plecanatide (brand name Trulance), also received FDA approval for CIC (Kamuda and Mazzola, 2018). These GUCY2C agonists are taken orally, and are highly specific to GUCY2C. Notably, the activity of oral linaclotide is restricted strictly to the intestine, with a bioavailability of 0.1%, limiting the effect of these oral agonists on GUCY2C outside of the intestinal lumen (Bryant et al., 2010).

In clinical trials, IBS-C and CIC patients treated with linaclotide and plecanatide experienced reduced abdominal pain (Castro et al., 2013; Sayuk et al., 2021). In animal models, even mice and rats without constipation reacted to GUCY2C agonists with analgesia (Eutamene et al., 2010; Boulete et al., 2018). Even more surprising, in a rat model of endometriosis oral linaclotide decreased abdominal pain (Ge et al., 2019). Similarly, bladder pain and dysfunction decreased in mice treated with oral linaclotide (Grundy et al., 2018). These results are unexpected given that GUCY2C is not found in the bladder, endometrium, or mesentery, and that GUCY2C agonists have no bioavailability outside of the intestine when administered orally. Thus, intestinal GUCY2C activation results in generalized visceral analgesia separate from its effects on secretion and constipation. Two mechanistic explanations for GUCY2C-induced analgesia have been proposed in the literature. In one model, GUCY2C induces cGMP accumulation in enterocytes, which is subsequently secreted basolaterally to signal in a paracrine fashion to visceral afferents. Evidence for this mechanism comes from studies showing that extracellular cGMP modulates sensory neuron activation, and inhibition of cGMP transporters in intestine decreases the analgesic effects of linaclotide (Brierley et al., 2022). For this hypothesis to be true, sensory neurons would need a receptor that responds to extracellular cGMP, which has yet to be discovered (Grundy et al., 2018). Another possible mechanism is that GUCY2C expressed in EECs (specifically neuropod cells) is responsible for neuromodulation of pain, and GUCY2C expressed in enterocytes is responsible for secretion driven by GUCY2C agonists. This bifurcated mechanism is supported by several observations. Indeed, mice without GUCY2C in neuropod cells are unresponsive to linaclotide analgesia; the secretory machinery is upregulated in enterocytes but downregulated in EECs; and EECs play a fundamental role in controlling visceral pain (Barton et al., 2023; Bayrer et al., 2023). This hypothesis too is incomplete, as the mediators of GUCY2C signaling both within EECs and between EECs and neurons remain unknown. It is possible that both hypotheses are true, and that EECs are the cells responsible for visceral pain modulation, but that the mediator of this interaction with peripheral nociceptors is extracellular (or even synaptic) cGMP. Further exploration of linaclotide’s effect on visceral pain could help develop a drug that is safe and effective for pain throughout the abdomen, while ameliorating unwanted side effects and avoiding narcotic pain relief.

## 7. Conclusion

**Figure 1:**
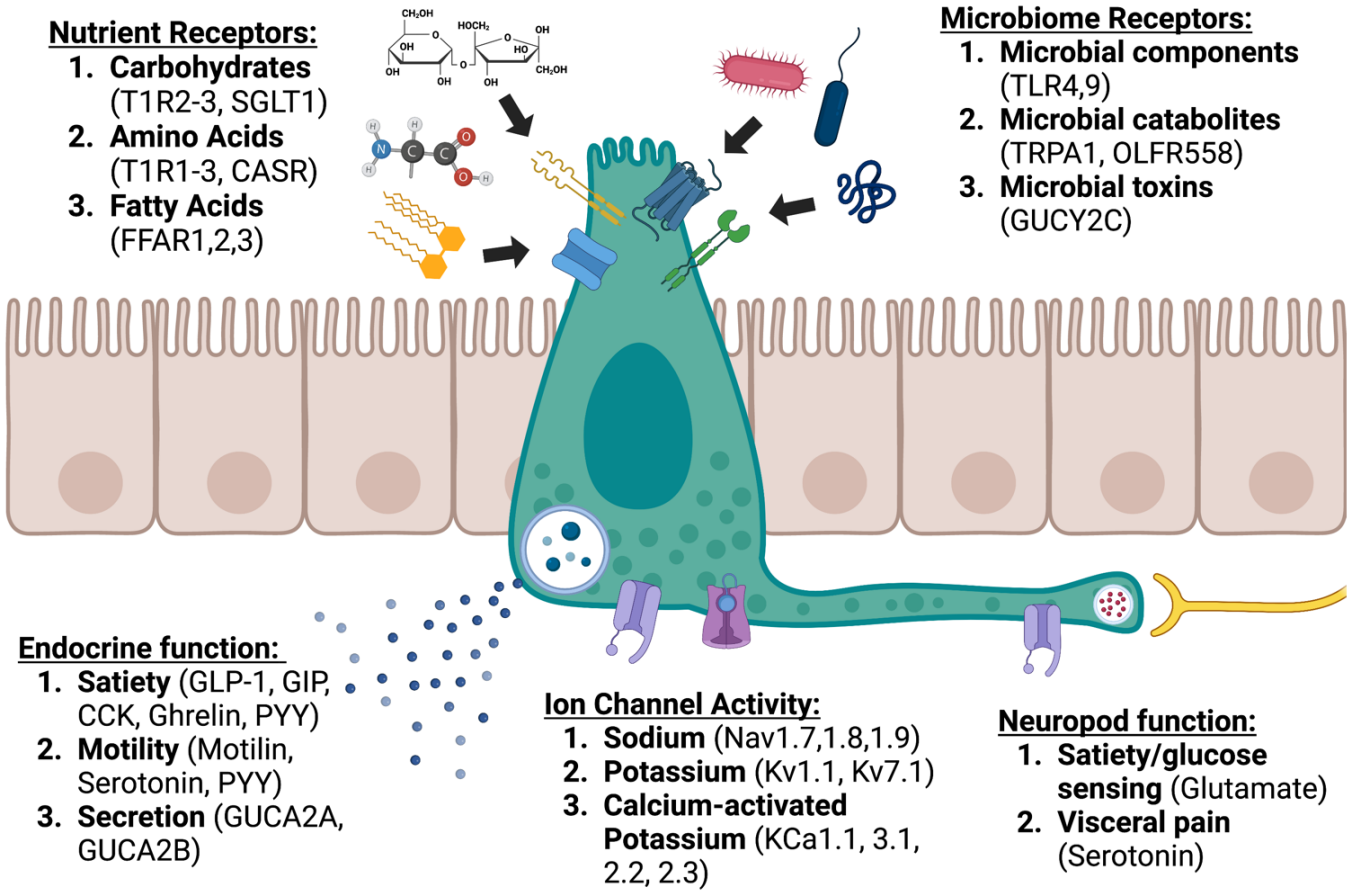
Enteroendocrine cells: sentinels of the gut brain axis (created with BioRender.com).

EECs are a chief component of how the body senses itself. Like proprioception, where the body senses position in space to coordinate movement, interoception allows the body to monitor dynamic processes like digestion and maintain homeostasis. EECs respond to a variety of luminal contents and perform complex integrated functions through intracellular signaling and careful modulation of ion channel dynamics. These processes allow the gut to send nuanced communications to the nervous system both directly through synapses and indirectly through endocrine and paracrine signaling (Figure 1). Though often these signals are not consciously perceived, the central nervous system organizes and responds to them, communicating back to the gut and EECs through the vagus nerve (Bonaz et al., 2018). We are just beginning to understand the delicate orchestration of this conversation, and certainly many more components than we have discovered are involved. We do know that disruption of this system is associated with severe GI dysfunction, including malabsorption, functional GI disorders, and obesity. Leveraging the role of EECs in the gut-brain axis led to development of safe and effective medications for some of these disorders. The success of pharmaceuticals like semaglutide and linaclotide brings renewed interest to the study of the gut-brain axis. This is underscored by the development of a triple hormone receptor agonist, retatrutide (agonist of GLP1R, GIP receptors, and glucagon receptors), by Eli Lily (Coskun et al., 2022). In a recently published phase 2 clinical trial, participants lost up to 24.2% body weight at 48 weeks on the 12 mg dose (Jastreboff et al., 2023). Impressively, 83% of participants who received the 12 mg dose lost at least 15% body weight by 48 weeks (Jastreboff et al., 2023). Retatrutide, like other GLP1R agonists also improved glycemic control, with an average hemoglobin A1c reduction of 2.02% with the 12 mg dose (Rosenstock et al., 2023). Unfortunately, like other GLP1R agonists, retatrutide also produced significant GI side effects in a large proportion of participants (35% in the 36 week study and 45% in the 48 week study at the 12 mg dose) (Jastreboff et al., 2023; Rosenstock et al., 2023). Further investigation of EECs and the gut-brain axis has the potential to impact not only diseases of the GI system but also devastating neurological diseases like Parkinson’s disease, Alzheimer’s disease, and stroke (Yu and Li, 2022; Zhou et al., 2023).

## Author contributions

JB: Conceptualization, Funding acquisition, Supervision, Writing – original draft, Writing – review & editing. AL: Writing – original draft, Writing – review & editing. TA: Writing – original draft, Writing – review & editing. AE: Writing – original draft, Writing – review & editing. MC: Writing – review & editing. SW: Writing – review & editing.

## Figures and Tables

**Table:** 

AP	Action potential
AgRP	Agouti related peptide
AITC	Allyl isothiocyanate
ARX	Aristaless-related homeobox
APECED	Autoimmune polyendocrinopathy-candidiasis-ectodermal dystrophy
APS-1	Autoimmune polyglandular syndrome type 1
AIRE	Autoimmune regulatory gene
CasR	Calcium sensing receptor
KCa	Calcium-activated potassium channel
CCKAR	CCK receptor
CCK	Choleycystokinin
ChrgA	Chromogranin A
cAMP	Cyclic AMP
cGMP	Cyclic GMP
DRG	Dorsal root ganglion
ECs	Enterochromaffin Cells
EECs	Enteroendocrine cells
ETEC	Enterotoxigenic *E. coli*
FFA	Free fatty acid
GI	Gastrointestinal
Ghrl	Ghrelin
GLP1	Glucagon-like peptide 1
GLP1R	Glucagon-like peptide 1 receptor
GIP	Glucose-dependent insulinotropic polypeptide
GIPR	Glucose-dependent insulinotropic polypeptide receptor
GFP	Green Fluorescent Protein
GUCY2C	Guanylin (GUCA2A), guanylyl cyclase C
STa	Heat stable enterotoxin
HFD	High fat diet
IBS	Irritable bowel syndrome
LPS	Lipopolysaccharides
NEUROG3	Neurogenin 3
Nts	Neurotensin
NO	Nitric Oxide
OLFR558	Olfactory receptor 558
PYY	Peptide YY
PDE	Phosphodiesterase
PPG	Preproglucagon
PCSK1	Proprotein convertase subtilisin/kexin type 1
PKA, PKC, PKG	Protein kinase A, C, G
Sct	Secretin
5-HT	Serotonin
SCFA	Short chain fatty acid
SGLT1	Sodium glucose transporter 1
SNARE	Soluble NSF Attachment Protein Receptor
Sst	Somatostatin
TLRs	Toll-like receptors
TRPA1	Transient receptor potential ankyrin 1
UC	Ulcerative colitis
GUCA2B	Uroguanylin
VGCC	Voltage gated calcium channel
Kv	Voltage-gated potassium channel
Nav	Voltage-gated sodium channel
WT	Wild type
